# Implementation science: a role for parallel dual processing models of reasoning?

**DOI:** 10.1186/1748-5908-1-12

**Published:** 2006-05-25

**Authors:** Ruth M Sladek, Paddy A Phillips, Malcolm J Bond

**Affiliations:** 1 Flinders University, Adelaide, South Australia

## Abstract

**Background:**

A better theoretical base for understanding professional behaviour change is needed to support evidence-based changes in medical practice. Traditionally strategies to encourage changes in clinical practices have been guided empirically, without explicit consideration of underlying theoretical rationales for such strategies. This paper considers a theoretical framework for reasoning from within psychology for identifying individual differences in cognitive processing between doctors that could moderate the decision to incorporate new evidence into their clinical decision-making.

**Discussion:**

Parallel dual processing models of reasoning posit two cognitive modes of information processing that are in constant operation as humans reason. One mode has been described as experiential, fast and heuristic; the other as rational, conscious and rule based. Within such models, the uptake of new research evidence can be represented by the latter mode; it is reflective, explicit and intentional. On the other hand, well practiced clinical judgments can be positioned in the experiential mode, being automatic, reflexive and swift. Research suggests that individual differences between people in both cognitive capacity (e.g., intelligence) and cognitive processing (e.g., thinking styles) influence how both reasoning modes interact. This being so, it is proposed that these same differences between doctors may moderate the uptake of new research evidence. Such dispositional characteristics have largely been ignored in research investigating effective strategies in implementing research evidence. Whilst medical decision-making occurs in a complex social environment with multiple influences and decision makers, it remains true that an individual doctor's judgment still retains a key position in terms of diagnostic and treatment decisions for individual patients. This paper argues therefore, that individual differences between doctors in terms of reasoning are important considerations in any discussion relating to changing clinical practice.

**Summary:**

It is imperative that change strategies in healthcare consider relevant theoretical frameworks from other disciplines such as psychology. Generic dual processing models of reasoning are proposed as potentially useful in identifying factors within doctors that may moderate their individual uptake of evidence into clinical decision-making. Such factors can then inform strategies to change practice.

## Background

The recent evolution of Evidence-Based Practice (EBP) in the 1990s has given cause to reconsider research into decision-making in relation to all health and allied health disciplines, but especially the practice of medicine. EBP is the process of using the best available research evidence combined with the practitioner's skills and patient's values to aid clinical decision-making. Surprisingly, despite the increasing availability of scientific evidence, gaps between this evidence and medical practice have consistently been reported [1], with often slow and haphazard uptake of new evidence [2]. There is now emerging interest in identifying and understanding such variations in practice, the barriers to changing existing clinical behaviour, and effective methods for changing medical practice. This field is referred to as implementation science. It has been argued that failures to implement the best evidence can result from either the lack of understanding, or lack of application of relevant theories from the social or educational sciences, and many popular strategies to improve the quality of healthcare have been chosen empirically, unguided by theory [3]. Most recently, it has been argued that a better theoretical base for understanding professional behaviour change is needed to support evidence-based changes in practice, and that there are a range of theories that need to be considered [2].

Theories relate to the individual (e.g., cognitive and educational theories), social interaction and context (e.g., social learning theory), and organisational and economic contexts (e.g., theories of innovative organisations) [4]. Our interest lies in considering a group of theories from within the psychological research tradition relating to the individual doctor. We suggest that parallel dual processing models of reasoning potentially are useful in identifying factors that influence the uptake of new evidence by individual doctors. Firstly we describe the nature of parallel dual processing models of reasoning, then discuss the uptake of best evidence by clinicians within the context of these, and go on to summarise some of the individual differences in cognitive processing that may influence the uptake of evidence by doctors. The implications of differences in thinking dispositions for implementation science and medical education are then considered.

## Discussion

### Models of reasoning

Several reviews have found strong support for the existence of dual processing models of reasoning [5-8]. Whilst different models use different terminology, it has been argued that there are strong family resemblances between the various theories [7]. Essentially all such models posit two cognitive modes of information processing that are in constant operation as humans reason. One mode has been described as experiential, unconscious, fast, associative, heuristic, tacit, quick, intuitive, recognition primed, implicit, automatic and acquired via biology or exposure. The other mode has been described as rational, conscious, deliberate, slow, rule-based, analytic, explicit, controlled, and acquired by cultural and formal tuition [7]. These two systems have generically been referred to elsewhere as System 1 and System 2, respectively. However, in this paper we use the terms 'experiential' and 'rational' to denote these two modes [9].

Dual processing models of reasoning have been conceptualised in two ways. First, reasoning can be *either-or*, where experiential processing is chosen in circumstances of low motivation; for example, when a judgement is considered relatively unimportant. Conversely, rational processing is chosen when the stakes are high. The Heuristic-Systematic Information Processing Model is an example of an either-or account, where a decision maker uses either simple decision rules (referred to as heuristic), or a systematic approach, with the choice being mediated, for example, by the degree of involvement the person has with the decision [10]. Such either-or models may not accommodate clinical decision-making well, because they position underlying motivation as the determinant of a person's processing mode. It would be difficult to argue, for example, that doctors making decisions in the experiential mode are less motivated to make correct diagnoses than those operating in the rational mode.

Second, these two reasoning systems may work in *parallel*. The Cognitive-Experiential Self Theory (CEST) is a parallel account, where both an experiential and rational system operate continuously in an integrated interaction [9]. This framework may be more appropriate for medical decision-making, as reasoning using the experiential mode operates regardless of the decision maker's level of motivation or the importance of a judgement. Within this model, all behaviour is considered to reflect the joint operation of both the experiential and rational modes. It has been suggested that there are five ways in which a judgement can be made within a dual processing model [11]:

 experiential mode judgement is endorsed by the rational mode,

 experiential mode judgement is insufficiently adjusted by the rational mode,

 experiential mode judgement is corrected (possibly over-corrected) by the rational mode,

 experiential mode judgement is identified as violating a rational rule and is blocked,

 no experiential mode judgement is made, so the rational mode calculates one.

The relationship between experiential and rational modes of reasoning has been shown to be influenced by a range of both dispositional (individual) and situational (environmental) factors. The corrective operations of the rational mode are known to be impaired by time pressure, involvement in a concurrent cognitive task, time of performing tasks compared to being a morning or evening person, and mood. The rational mode of operating has been shown to be positively correlated with intelligence, need for cognition (tendency to engage in and enjoy thinking) and exposure to statistical training [11]. The experiential mode has been shown to be influenced by faith in intuition [9].

### Implementation of evidence and dual processing models of reasoning

If, as has been argued elsewhere, the time has come to consider various theoretical bases from other disciplines for evidence-based implementation strategies in medicine [2], there would appear to be a prima-facie case for considering parallel dual processing models of reasoning. Within such models, the uptake of new research evidence can be represented by the activities of the rational mode of reasoning. For example, the decision to include a new treatment regimen based on a newly published evidence-based guideline for an individual patient is conscious, explicit and intentional. On the other hand, existing clinical practice can be positioned in the 'experiential' mode: well rehearsed judgements based on years of clinical experience may be viewed as unconscious, automatic, reflexive and swift. Changing practice, therefore, would require activation of the rational mode of reasoning to work in certain ways, as noted earlier [11]. In other words, changing an individual doctor's clinical practice (an experiential mode judgement) would require activation of their rational mode to consciously adjust or override that existing judgment. As well rehearsed judgments over time are thought to change from the rational to experiential mode of reasoning, it may be that more experienced clinicians are likely to be slower to change long-standing, often practiced judgments.

Whilst our interest is the implementation of new evidence into existing clinical practice, it is also interesting to consider *error *within this model, which could reflect the under- or over-correction activities of the rational mode in relation to the experiential mode. More than 30 years of research within the heuristics and biases tradition has demonstrated systematic patterns of error in reasoning and thinking tasks [12].

Recently this body of work has been reconsidered alongside developments in parallel dual processing models of reasoning. Systematic error has been postulated as reflecting error within the experiential mode [11]. While the theoretical interpretations of this error are controversial [12,13], the practical implications of these errors are most important in terms of improving the quality of clinical decision-making. More than 40 specific cognitive biases and their implications for clinical practice have now been identified, and it has been argued that most diagnostic errors are the result of cognitive errors, given that the process of diagnosis largely depends on a clinician's thinking [14,15]. The nature of these biases vary but commonly involve heuristics, considered to be 'quick natural assessments' based on some attribute of a decision. For example, a decision can be biased by an initial affective reaction (affect heuristic), how easily similar circumstances are recalled (availability heuristic), or how representative the circumstance is to a recognised stereotype (representativeness heuristic) [12]. Whilst often these heuristics work well, they may on occasion lead to sub-optimal decision-making. Such heuristic biases may lead to diagnostic error, including both missed diagnoses and misdiagnoses, with potential negative consequences for patients. Table 1 provides illustrative medical examples of these heuristics [16-19].

**Table 1:** Illustrative medical examples of selected heuristics

**Heuristics**	**Examples**
*Availability*: Estimating the likelihood of an event (X) by the ease with which instances of X come to mind, i.e., how available they are.	Recent experiences caring for patients with bacteremia were associated with doctors' higher estimated probabilities that hospital inpatients (for whom blood cultures had been taken) had bacteremia [16].
*Representativeness*: Where estimating the likelihood of an event (X) is mediated by the degree to which it represents the class to which X belongs.	Drives the diagnostician toward looking for prototypical manifestations of disease: "If it looks like a duck, walks like a duck, quacks like a duck, then it is a duck." Yet restraining decision-making along these pattern-recognition lines leads to atypical variants being missed [17]. It inappropriately ignores, for example, prior probabilities [18].
*Affect*: Where an initial affective reaction biases the resultant decision.	When toxicologists were asked to assess the risk associated with a very small exposure to 30 chemical items, degree of risk was mediated by their assessment of how 'bad-good' each chemical was [19].

If consideration of new research evidence is positioned as a rational mode function, and current practice as an experiential mode function, then the factors which influence the relationship between these two modes may be of importance in understanding the uptake of new evidence in medical practice. In other words, those factors which have been shown to restrict or facilitate the operations of the rational mode may influence the uptake of new evidence. As noted earlier, research has demonstrated that both situational and dispositional factors are influential. External situational factors such as the social, economic, administrative and organisational context have been widely identified as barriers to the uptake of evidence, as have dispositional characteristics such as knowledge, skills, attitudes, values and personality [4]. However, some of these dispositional factors, namely those characteristics of the individual which are relatively stable over time, have been largely ignored in evidence implementation strategies in healthcare.

Despite acknowledging that medical decision-making occurs in a complex social environment with multiple influences and decision makers, it remains true that an individual doctor's judgement still retains a key position in terms of diagnostic and treatment decisions for individual patients. It is therefore relevant to consider how the individual dispositions of doctors may influence decision-making and clinical practice. As has been noted, it would actually be surprising if personality was not related to medical decision-making [15]. Whilst individual differences in personality are known to influence various aspects of patient encounters, such as communication and interpretation of patient behaviour, of importance to the current discussion are the particular aspects of personality *which relate to thinking*. That is, how doctors reason, and individual differences between doctors in reasoning are relevant considerations in discussions of medical decision-making.

### Thinking dispositions

Within reasoning research, there has been a predominant focus on intelligence. For example, an extensive review of individual differences in reasoning is dominated by a discussion of research relating to cognitive capacity [7]. In this context intelligence is typically measured by the Scholastic Aptitude Test (SAT) or other measures of general intelligence. However, intelligence is not of direct interest to our discussion in relating this model to doctors and practice change because we assume that, in general, all doctors are highly intelligent, and indeed, most probably reflect a small, attenuated range of higher Intelligent Quotient (IQ) scores.

However, it has been argued that reasoning may represent a range of dispositions that are quite distinct from intelligence [20,21]. Other individual differences in cognitive processing (as opposed to intelligence) also may be important and are of interest to the present discussion. These have been referred to variously, including thinking dispositions, thinking styles, or styles of epistemic regulation, but the differing terms are used in similar ways to denote 'relatively stable psychological mechanisms and strategies' [13]. The term 'thinking dispositions' is used in this paper. Whilst cognitive capacity has been associated with an algorithmic level of cognition (ie, computational processes), it has been suggested that individual differences in cognitive processing influence the intentional level of analysis, that is to say a particular level of analysis in cognition which is thought to reflect an individual's goals, values and beliefs [13]. This would seem then, particularly relevant for any discussion on the explicit use of evidence in medical practice, which by its nature incurs intentional analysis.

Several candidate constructs may be usefully considered. 'Actively open minded thinking' [22] (measured by composite scores from scales for constructs such as dogmatism and categorical thinking) has been found to predict biased thinking independent of cognitive capacity [20]. 'Need for cognition' refers to individual differences in the tendency to engage in and enjoy thinking [23]. It has been associated with the rational mode of reasoning and shown to be related to, but not the same as intellectual ability. As an analogy, a person's motivation to engage in physical activities is related to, but not the same as physical ability [24]. Need for cognition also has been incorporated into the Rational-Experiential Inventory instrument, which additionally includes a related but independent construct termed 'faith in intuition' that has been shown to influence the experiential mode of reasoning [9].

Cognitive style may be another construct of interest as it appears at least conceptually similar to the need for cognition and faith in intuition. Cognitive style refers to the way in which an individual takes note of the surroundings, seeks meaning and becomes informed [25]. One popular measure of cognitive style is an individual's preferred modes of information-intake and decision-making, as measured by two of the polar preference scales of the Myers-Briggs Type Indicator (MBTI): sensing-intuiting (S-N), and thinking-feeling (T-F) [26]. The four possible preference types according to these scales (NT, NF, ST, and SF) have been associated with need for cognition [27], and it is therefore conceivable that they, too, may measure important individual differences in reasoning. Indeed, it has been noted that future research could usefully relate need for cognition and faith in intuition to the two dichotomies of the Myers-Briggs Type Indicator measuring cognitive style [28].

There are likely to be other constructs of interest, but the foregoing have already accumulated some research evidence in support of their independence from intelligence, and possible influence on reasoning modes, albeit limited. Whilst these individual differences can be seen as important in understanding reasoning, they also may be important in terms of attitudes to EBP, which in turn may moderate the uptake of evidence. The directional influences of these constructs remain to be demonstrated. On one hand, those with a higher need for cognition may be more open to considering new knowledge, and thus may have more favourable attitudes to EBP. Those with a higher need for faith in intuition may be more affectively attracted to retaining existing practices, with which over time they have been 'satisfied.' However, these influences also might be in the opposite direction. Someone with higher faith in intuition might have more favourable attitudes toward EBP because of the strong emotional appeal of being 'right' and 'proper' to integrate the best research evidence into practice. However a person with a higher need for cognition might have less favourable attitudes because they have thought through the limitations of such an approach, for example, of generalising the results of large trials to their individual patient, who has different characteristics compared with those included in the original trial.

Whilst there is a considerable amount of research into medical decision-making, it is worth noting that there is little research investigating the role of individual differences in cognitive processing and clinical decision-making. Research using the Myers-Briggs Type Indicator has focused largely on overall type (not specifically cognitive style), demonstrating that certain types are attracted to certain specialties [29]. More recently, differences in type between patients and doctors have been explored, suggesting that understanding differences in type can improve clinician-patient communication [30]. In a small, underpowered, study an association was found between type and laboratory test ordering for hypertensive patients [31]. In more than 100 studies that have validated the need for cognition as a distinct and measurable construct using the Need for Cognition Scale, nearly all used general undergraduate students or community members, with no published studies having used medical practitioners as participants [24].

### Implications for change strategies and medical education

Empirically-driven strategies to change practice in accordance with new evidence yield some success, but changes are typically modest [1]. Whilst empirical 'top-down' strategies are based on 'what works,' they still leave unanswered the question – "why does it work?" Answering this question could ultimately inform the generalisability of any study investigating the implementation of evidence into practice. Researchers need to underpin studies investigating evidence implementation with explicit theoretical rationales, something that has been scarcely done to date [2,3]. Parallel dual-processing models of reasoning offer a framework for understanding the decision-making of individual doctors. Understanding the target group for change is an important precursor to the selection of change strategies in the effective organisation of evidence implementation [4].

Considering such models of reasoning has several implications. First, if thinking dispositions are important, these need to be investigated further to determine their nature, how they vary between individuals, how they affect decision-making and clinical behaviour, and their interactions with other influences. Such other influences might include variables such as conditions of uncertainty. Relationships between thinking dispositions and clinical decision-making may initially be investigated theoretically using surrogate measures of clinical practice (e.g., clinical scenarios), although ultimately these relationships need to be demonstrated in actual practice.

Second, thinking dispositions may not only influence the direct process of clinical decision-making. In a broader sense, they may also influence receptivity to messages and different styles of message delivery. As an example, consider an evidence-based guideline within Cognitive-Experiential Self Theory. Reality is thought to be encoded in the experiential system in images, metaphors and narratives, as opposed to abstract symbols, words and numbers in the rational system [24]. If there are individual preferences for one mode over the other, then implementation strategies would best target both modes. For example, a guideline might be written to include both case studies (targeting the experiential system) and a full verbal and numerical exposition of the evidence (targeting the rational system). In terms of the implementation of such a guideline, it may be that use of opinion leaders to implement the guidelines might appeal to those who prefer the experiential system, whereas peer debate at conference meetings might appeal to those preferring the rational system. The direct implication is that implementation strategies need to utilise multi-faceted approaches in both the design of presenting evidence and the strategies to encourage uptake of that evidence.

Third, it may be true that doctors with different thinking dispositions are preferentially attracted to various specialties [29]. If the nature of such preferences were understood, then strategies could be designed to accommodate the prevailing styles associated with a particular specialty. Fourth, the underlying premise of parallel, dual processing models of reasoning is that neither processing mode is superior, and that both contribute to optimal decision-making. Whilst the experiential system might be adaptive, it will be deficient for decisions requiring rational analysis. Conversely, it would be highly inefficient to rely solely on the rational system for all decisions [32]. There may be an adaptive explanation for doctors' resistance to change in practice, and indeed, such resistance may be valuable. Whilst this might be dispositional, it also may be learned through previous experience. Medical history is littered with treatments which were believed to be beneficial, but were ultimately found to cause harm. Consider the assumption behind implementation science: that practice more in accordance with evidence will maximise population health outcomes. There is an alternative position: healthy scepticism to change will minimalise harm. For example, perhaps the same reluctance of a general practitioner (GP) to change his or her clinical practice in response to emerging evidence is also manifested in his/her scepticism of drug marketing campaigns. The challenge for implementation science is to understand the ways in which each processing mode both potentially enhances and compromises optimal clinical decision-making.

Ultimately, implementation strategies that focus solely on situational factors may be as inappropriate as those focused solely on dispositional factors. However there is a limit to the size and nature of implementation strategies that can be undertaken, restrained by resources such as time and funding. Some have argued that theories of relevance to implementation research are most likely to include only modifiable variables [2]. Perhaps this is too narrow. Any theory of behaviour or cognition potentially will be of relevance to implementation science. Models that fail to include human traits will be limited when it comes to the design of strategies and the interpretation of why and under what circumstances they lead to changed practice.

Consider a hypothetical model which effectively predicts what is known as 'external information search,' such as proactively seeking information from an evidence-based guideline on the Internet. We might know, and successfully predict, that a certain percentage of GPs with broadband access (a modifiable variable) will use the Internet to refer to an evidence-based guideline (target behaviour) once a week during a patient encounter. We might also know that a 10 minute training session on how to find such guidelines will lead to an increased sustained rate of such behaviour (a second modifiable variable). We could then target both variables – increased broadband access and a brief directed educational intervention – as a strategy to improve compliance with the best evidence, and find that we raise the percentage of those access rates. Let it also be assumed that it is true that a higher need for cognition (a non-modifiable, stable variable) also predicts the percentage of GPs likely to initiate an external information search. Without knowing the influence of need for cognition, we would not have an accurate picture of the number of GPs likely to be open to change. Thus, understanding stable variables contributes to defining the potential for change, which may be important for considering and comparing implementation strategies.

There are direct implications of individual differences in thinking dispositions for medical education from undergraduate through postgraduate training. Doctors need to be trained not only in formal decision-making, but also in critical thinking and problem solving. They need to develop their meta-cognitive skills – the ability to think about how they think – and recognise their vulnerability to a multiplicity of biases [17]. Additionally, doctors need to understand their own unique preferences in thinking styles, and how these may contribute to their practice of medicine and development of clinical behaviours over time. Insight, whilst important, should not be the sole goal of training. There is a need to research which strategies effectively address counter productive tendencies, and to provide trainees with a working knowledge of these [17]. Such strategies are referred to as 'debiasing.'

It has been proposed elsewhere that humans are capable of meta-cognitive skills (the ability to think about how we think) and that we can create cognitive forcing strategies to inoculate against known biases in our thinking [17]. The example given is that the most commonly missed fracture is the 'second one' because doctors tend to stop their search for information too early in the diagnostic process. Such premature closure in a search strategy is known as 'satisficing' and can occur in any part of the diagnostic process in clinical practice. This potential pitfall in thinking could be overcome by teaching doctors a generic cognitive forcing strategy in the emergency room: once you obtain a positive finding or fail to find an expected result, begin a secondary search. Such a strategy is not just relevant for the one clinical problem of fractures; it is directly applicable to a host of other presentations, such as looking for co-ingestants in self-poisoning, and failing to look for a second foreign body [17].

## Summary

We have initially considered generic, parallel dual-processing models of reasoning. The next step is the identification of a specific theory that can be evaluated for its relevance and appropriateness to medical practice, such as the Cognitive-Experiential Self Theory. Descriptive information on the type and variation of individual differences in cognitive processing amongst doctors needs to be identified. This may include thinking styles, need for cognition, faith in intuition, and possibly cognitive style. Such differences will need to be predictably related to clinical judgements and real life practice, as well as attitudes to EBP. Then effective change strategies based on this knowledge can be contemplated. There may be preferred change strategies depending on the cognitive processing profile for different groups of doctors.

It is imperative that change strategies in healthcare consider relevant research from other disciplines, such as psychology, and look towards identifying an appropriate theoretical basis. Empirical strategies, whilst no doubt necessary, are not solely sufficient. We have considered parallel dual processing models of reasoning and positioned the uptake of new evidence as a rational mode function, and clinical experience as an experiential mode function. By viewing these two components of medical decision-making within this model, we infer that the same influences that have been shown to influence the interaction of both modes of reasoning in other settings, may moderate the uptake of evidence in clinical practice. We have particularly focused on the role of individual differences in cognitive processing. Whilst most research relates to individual differences in intelligence, some emerging research points to other differences in cognitive processing, such as thinking styles, need for cognition, and faith in intuition. A better understanding of individual differences between doctors on these constructs may ultimately contribute to the design of strategies to improve uptake of new research evidence by doctors.

## Competing interests

RS is a PhD research scholar supported by the National Institute of Clinical Studies (NICS), Australia's national agency for closing the gaps between evidence and practice in health care.

## Authors' contributions

All authors made significant contributions to the conception of the paper. RS drafted the manuscript, and PP and MB revised it critically for its intellectual content. All authors read and approved the final manuscript.
